# Supplementary material for: Influenza A virus is transmissible via aerosolized fomites
Source: Nat Commun. 2020 Aug 18;11:4062. doi: 10.1038/s41467-020-17888-w (PMC7435178; doi:10.1038/s41467-020-17888-w)
Supplement: Supplementary file 1 — Supplementary Information [file 41467_2020_17888_MOESM1_ESM.pdf]

Supplementary Information:
Influenza A virus is transmissible via aerosolized fomites

Asadi et al.

Supplementary Fig. 1. APS measurement cage used to measure particle generation by mobile guinea pigs on different types of beddings.

Supplementary Fig. 2. Representative particle emission rates from an awake mobile and an anesthetized guinea pig.

Supplementary Fig. 3. Size distribution of airborne particles emitted from different sources.

Supplementary Fig. 4. Custom-built equipment for APS quantification of respiratory particulates produced by anesthetized and euthanized guinea pigs.

Supplementary Fig. 5. Custom-built cage unit for modeling airborne influenza virus transmission in guinea pigs.

Supplementary Fig. 6. Virus viability on the bodies and in the environment of Pan99-immune guinea pigs painted with Pan99 stock virus.

Supplementary Fig. 7. Aerosolized fomite generation from influenza virus-contaminated paper tissues and collection with a bioaerosol sampler.

Supplementary Discussion 1. Estimating the amount of infectious virus aerosolized by rubbing paper tissues.

a b c

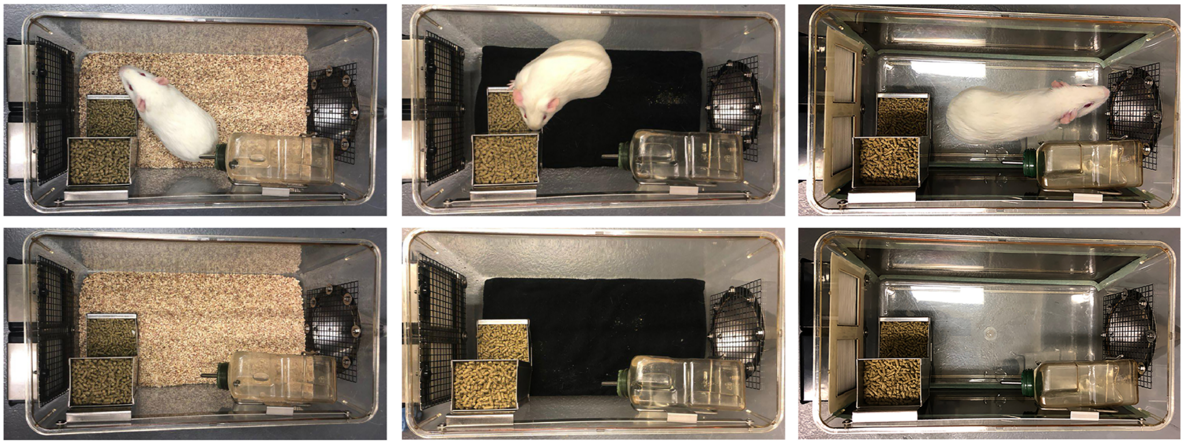

Supplementary Fig. 1. APS measurement cage used to measure particle generation by mobile guinea pigs on different types of beddings. Time-lapse images taken during APS quantification of guinea pig-generated airborne particulates in an airtight, HEPA-filtered polycarbonate cage, shown with (**a**) dried corncob granules (CC) bedding, (**b**) polar fleece-covered absorbent pad (PF) bedding, and (**c**) no bedding on the cage floor. Food and water were also provided during measurements.

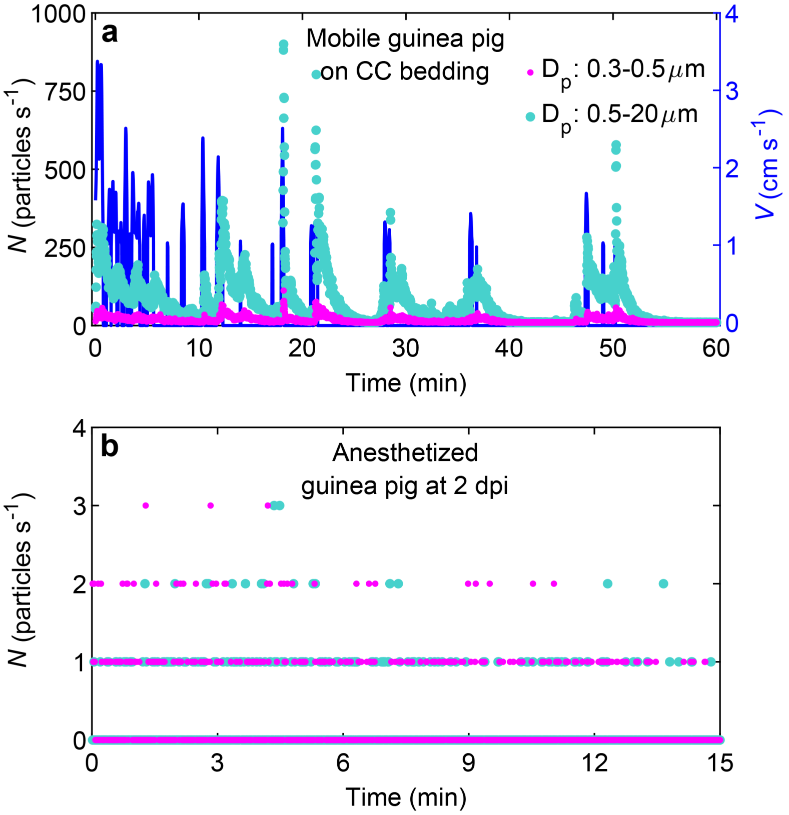

Supplementary Fig. 2. Representative particle emission rates from an awake mobile guinea pig and an anesthetized guinea pig. (**a**) Instantaneous particle emission rate (left axis), and instantaneous guinea pig movement velocity (right axis, blue line), versus time for a mobile guinea pig on dried corncob granule (CC) bedding. (**b**) Particle emission rate for an anesthetized guinea pig 2 days post-inoculation (dpi). Magenta and turquoise markers in (**a**) and (**b**) show the small (D_p: 0.3-0.5 μm) and large (D_p: 0.5-20 μm) particles, respectively. Source data are provided as a Source Data file.

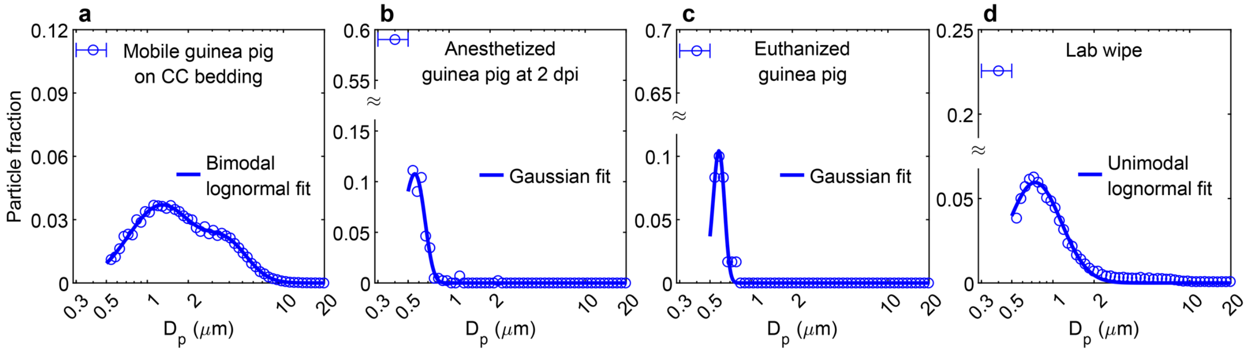

Supplementary Fig. 3. Size distribution of airborne particles emitted from different sources. (**a**) An awake, mobile guinea pig in the APS measurement cage with dried corncob granule (CC) bedding (Supplementary Fig. 1a, top image). (**b**) An anesthetized guinea pig at 2 days post-inoculation (dpi), and (**c**) a euthanized guinea pig inside aluminum sleeve placed in the APS measurement cage (Supplementary Fig. 4d). (**d**) Crumpling and rubbing a lab wipe in front of a stainless-steel funnel attached to the APS (Fig. 4a and Supplementary Movie 2). The left-most data point in each plot shows the fraction of particles counted in the 0.3 to 0.5 μm bin, which cannot be further size-discriminated. The whiskers represent the width of the bin (0.3 to 0.5 μm). Source data are provided as a Source Data file. APS: aerodynamic particle sizer.

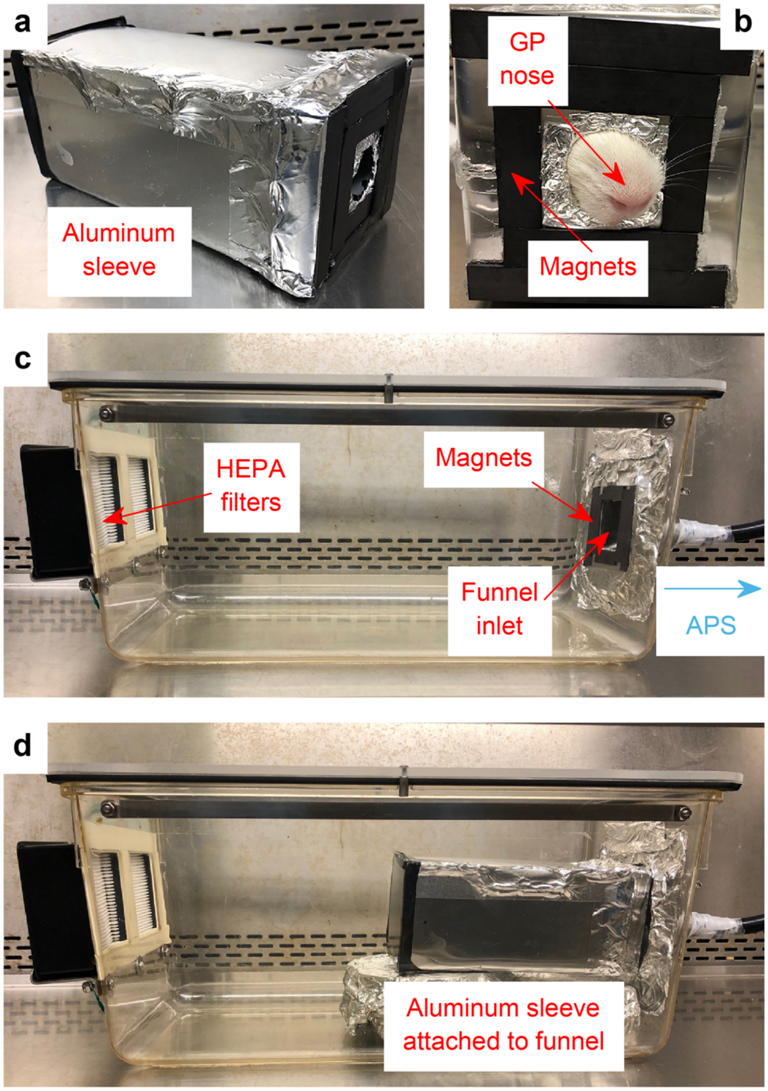

Supplementary Fig. 4. Custom-built equipment for APS quantification of respiratory particulates produced by anesthetized and euthanized guinea pigs. (**a**) Static-dispersing aluminum sleeve for containment of non-respiratory particulates. (**b**) An anesthetized guinea pig inside the aluminum sleeve with only its nose exposed. Magnets attach the aluminum sleeve to the funnel inlet inside the measurement cage. (**c**) The HEPA-filtered, airtight cage has a stainless-steel funnel mounted to the cage wall, which connects by conductive tubing to the APS. (**d**) The aluminum sleeve containing the anesthetized guinea pig is attached, nose-first, by magnets to the APS funnel inlet for respiratory particulate characterization. APS: aerodynamic particle sizer.

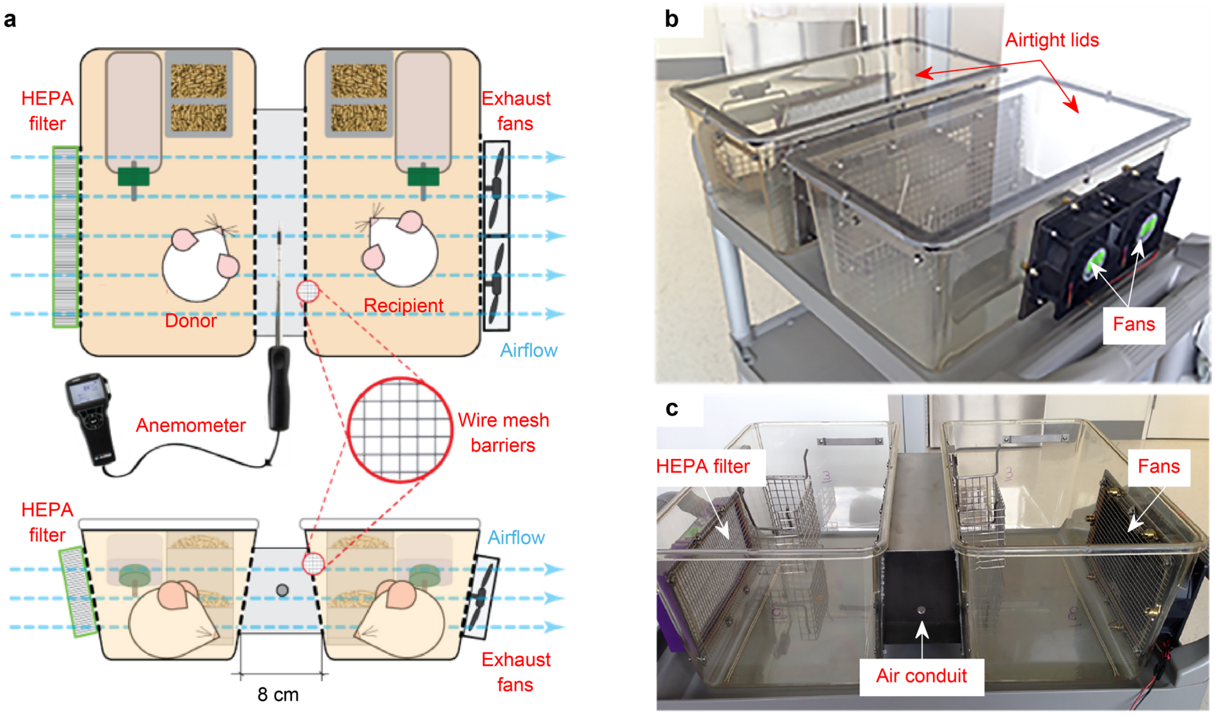

Supplementary Fig. 5. Custom-built cage unit for modeling airborne influenza virus transmission in guinea pigs. Schematic (**a**) and photographs (**b**, **c**) of caging used for airborne influenza virus transmission experiments in guinea pigs. Each transmission pair -- a virus donor and a virus recipient -- occupies a single cage unit, consisting of two standard polycarbonate cages joined together by a stainless-steel air conduit. Wire-mesh barriers on either side of the air conduit prevent contact between guinea pigs. Air is drawn unidirectionally, from donor guinea pig upstream to recipient guinea pig downstream, by two adjustable-speed fans that exhaust air outward from the recipient's cage. Air enters the cage unit through a HEPA-filtered inlet in the cage housing the virus-donor animal. Airflow speed can be regulated with a speed controller and a hot-wire anemometer probe, which measures and records air velocity, temperature, and RH in the center of the air conduit. See Methods for further details.

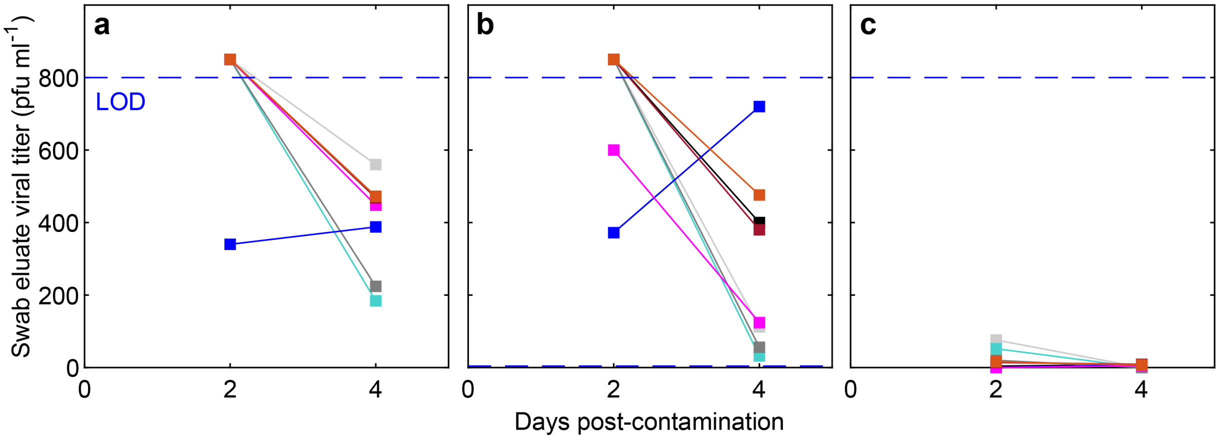

Supplementary Fig. 6. Virus viability on the bodies and in the environment of Pan99-immune guinea pigs painted with Pan99 stock virus. Swab samples, taken on days 2 and 4 post-contamination from the (**a**) fur, (**b**) ears, and (**c**) cages of Pan99-immune and -contaminated donor guinea pigs, were eluted into PBS supplemented with antibiotics and titrated by plaque assay. Horizontal dashed lines indicate the upper LOD (800 pfu ml^{-1} of swab eluate); the lower LOD (4 pfu ml^{-1}) is not shown. Titers shown above were performed during two of the three transmission experiment replicates represented in Fig. 3. One swab per area (fur, ears, paws, and cages) was taken per guinea pig per time point, and one plaque assay was performed from each swab eluate. Source data are provided as a Source Data file. LOD: limit of detection; pfu: plaque-forming units.

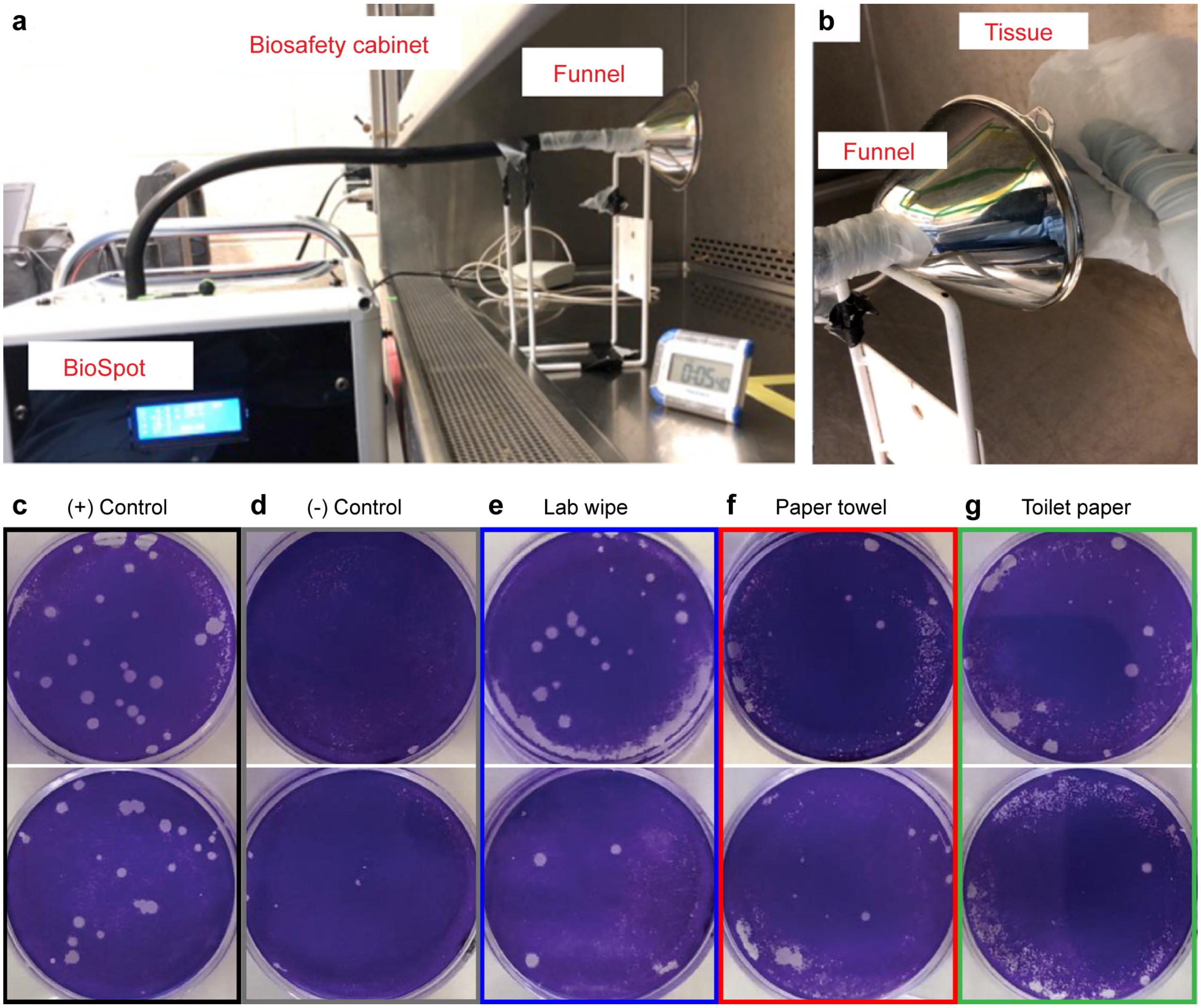

Supplementary Fig. 7. Aerosolized fomite generation from influenza virus-contaminated paper tissues and collection with a bioaerosol sampler. (**a, b**) Experimental set up. The BioSpot bioaerosol sampler draws air at 8 L min^{-1} through alternating warm and cold zones, enlarging airborne particulates by water condensation. Water-laden particulates impinge onto collection medium at the base of the air stream. (**c-g**) Plaque assays of the BioSpot collection media from air sampling of nebulized virus stock (positive control, **c**), virus-free air (negative control, **d**), and manual rubbing of a virus-contaminated lab wipe (**e**), paper towel (**f**), or toilet paper (**g**). One biological replicate of the positive control was performed, with two technical replicates (plaque assays) from the biological replicate. Both technical replicates are shown in (a). Two biological

replicates of the negative control were performed, with one technical replicate (plaque assay) from each. Both are shown in (b). Two biological replicates were performed with lab wipe and paper towel. Two technical replicates (plaque assays) were performed from each biological replicate. A technical replicate from each of the two biological replicates are shown in (e) and (f). Only one biological replicate was performed with toilet paper because it was too fragile to withstand 8 minutes of manipulation; both technical replicates of that biological replicate are shown in (g).

Supplementary Discussion 1. Estimating the amount of infectious virus aerosolized by rubbing paper tissues.

The paper tissues were contaminated with 3.6×10^5 pfu of virus on each individual tissue. To estimate the number of pfu actually aerosolized, we note that the APS detected instantaneous particle emission rates up to 900 particles per second (in the range of 0.3-20 μm). A conservative over-estimate is that 8 minutes of rubbing yielded on the order of 500,000 particles. From the particle size distribution, the geometric mean diameter on a mass basis (i.e., of the average diameter cubed) is 7.3 μm. Assuming the average density of the cellulosic fiber particles is 1.5 g cm^{-3}, then we estimate that during one tissue rubbing experiment only a total of 1.5×10^{-4} grams of the tissue were aerosolized:

$$\text{Total number of aerosolized particles} = \text{particle emission rate} \times \text{crumpling time}$$

$$= 900\ \frac{\text{particles}}{\text{s}} \times 480\ \text{s} = 432{,}000\ \text{particles} \cong 500{,}000\ \text{particles} \tag{1}$$

$$\text{Total volume of aerosolized particles} = \text{Total particles generated} \times \text{average particle volume}$$

$$= 500{,}000\ \text{particles} \times \frac{4}{3}\pi(0.000365\ \text{cm})^3 = 1.02 \times 10^{-4}\ \text{cm}^3 \tag{2}$$

$$\begin{gathered}\text{Total mass of aerosolized particles}\\ = \text{Total volume of particles} \times \text{average density of paper fiber}\\ = (1.02 \times 10^{-4}\ \text{cm}^3) \times 1.5 \frac{\text{g}}{\text{cm}^3} = 1.5 \times 10^{-4}\ \text{g}\end{gathered} \tag{3}$$

In comparison, a single Kimwipe paper tissue (21.33 cm × 11.17 cm) weighs 0.47 grams. Thus, only 0.032% of the original tissue mass was aerosolized into the collector. Assuming the virus was evenly distributed on the mass of the tissue, then of the original 3.6×10^5 pfu only a total of 115 pfu were actually aerosolized into the collector:

$$\text{Total pfu collected} = \text{Total pfu applied per tissue} \times \text{fraction of tissue mass aerosolized}$$

$$= (3.6 \times 10^5\ \text{pfu}) \times 0.032\% = 115\ \text{pfu} \tag{4}$$

This estimate yields a predicted emission rate of 14 pfu min^{-1} of tissue manipulation; the remainder of the virus stayed behind with the tissue. Since we observed on the order of 8 to 40 pfu in the plaque assays per tissue rubbing experiment (1 to 5 pfu collected per minute of air sampling), this estimate suggests that the BioSpot collector captured a sizable fraction of the viable virus. This estimate also suggests that roughly 1 out of 4,300 particles carried a pfu of virus.

Note that this estimate assumes that the virions were evenly spread out through the entire mass of the tissue paper, but the relative penetration of the virions into both the inter-fiber and intra-fiber pores of the paper during capillary imbibition of the liquid media possibly plays a role. In other words, a higher fraction of the virions might be located at the surface of the fibers, and thus

more readily aerosolized upon friction. Further research on the microscopic scale would be necessary to provide deeper insight on the virion distributions following capillary imbibition into fibrous media and the subsequent virion response to tribological forces.
