# Supplementary material for: Influenza A virus is transmissible via aerosolized fomites
Source: Nat Commun. 2020 Aug 18;11:4062. doi: 10.1038/s41467-020-17888-w (PMC7435178; doi:10.1038/s41467-020-17888-w)
Supplement: Supplementary file 3 — Description of Additional Supplementary Information [file 41467_2020_17888_MOESM3_ESM.pdf]

Description of Additional Supplementary Files

File Name: Supplementary Movie 1

Description: An infected guinea pig grooming. Pan99- infected guinea pig grooms itself, potentially facilitating virus spread to its body and environment.

File Name: Supplementary Movie 2

Description: Crumpling and rubbing paper tissue. A gloved hand rubbing a paper tissue in front of a stainless-steel funnel attached to an APS.
