# Supplementary material for: Influenza A virus is transmissible via aerosolized fomites
Source: Nat Commun. 2020 Aug 18;11:4062. doi: 10.1038/s41467-020-17888-w (PMC7435178; doi:10.1038/s41467-020-17888-w)
Supplement: Supplementary file 6 — Reporting Summary [file 41467_2020_17888_MOESM6_ESM.pdf]

nature research

Corresponding author(s): Bouvier, Nicole M.

Last updated by author(s): Jul 7, 2020

Reporting Summary

Nature Research wishes to improve the reproducibility of the work that we publish. This form provides structure for consistency and transparency in reporting. For further information on Nature Research policies, see Authors & Referees and the Editorial Policy Checklist .

Statistics

For all statistical analyses, confirm that the following items are present in the figure legend, table legend, main text, or Methods section.

n/a	Confirmed	
☐	☒	The exact sample size (n) for each experimental group/condition, given as a discrete number and unit of measurement
☐	☒	A statement on whether measurements were taken from distinct samples or whether the same sample was measured repeatedly
☐	☒	The statistical test(s) used AND whether they are one- or two-sided<br>*Only common tests should be described solely by name; describe more complex techniques in the Methods section.*
☒	☐	A description of all covariates tested
☒	☐	A description of any assumptions or corrections, such as tests of normality and adjustment for multiple comparisons
☐	☒	A full description of the statistical parameters including central tendency (e.g. means) or other basic estimates (e.g. regression coefficient) AND variation (e.g. standard deviation) or associated estimates of uncertainty (e.g. confidence intervals)
☒	☐	For null hypothesis testing, the test statistic (e.g. F, t, r) with confidence intervals, effect sizes, degrees of freedom and P value noted<br>*Give P values as exact values whenever suitable.*
☐	☒	For Bayesian analysis, information on the choice of priors and Markov chain Monte Carlo settings
☒	☐	For hierarchical and complex designs, identification of the appropriate level for tests and full reporting of outcomes
☐	☒	Estimates of effect sizes (e.g. Cohen's d, Pearson's r), indicating how they were calculated

Our web collection on statistics for biologists contains articles on many of the points above.

Software and code

Policy information about availability of computer code

Data collection	Data were collected with the following commercially available or open-source software:<br>Time-lapse photographic recording of guinea pig movement : iSpy (64-bit), version 7.2.1.0<br>Video recording of tissue crumpling experiment: MATLAB, version R2019a (MathWorks, Inc.)<br>Video recording of guinea pig grooming: iPhone 8 (Apple Inc.)<br>APS data collection: Aerosol Instrument Manager (AIM) software, version 9.0.0.0 (TSI Inc.)<br>Nasal wash and environmental swab virus titer data collection: Excel for Mac 2011 version 14.7.3 (Microsoft Corporation)
Data analysis	Data were analysed in MATLAB (version R2019a, MathWorks, Inc.) and R (version 3.6.3, R Foundation for Statistical Computing) and were graphed in MATLAB.

For manuscripts utilizing custom algorithms or software that are central to the research but not yet described in published literature, software must be made available to editors/reviewers. We strongly encourage code deposition in a community repository (e.g. GitHub). See the Nature Research guidelines for submitting code & software for further information.

Data

Policy information about availability of data

All manuscripts must include a data availability statement. This statement should provide the following information, where applicable:
- Accession codes, unique identifiers, or web links for publicly available datasets
- A list of figures that have associated raw data
- A description of any restrictions on data availability

Figures 1, 2, 3, and 4, and Supplementary Figures 2, 3, and 6 have associated raw data. Source data are available from the corresponding author upon reasonable request.

Field-specific reporting

Please select the one below that is the best fit for your research. If you are not sure, read the appropriate sections before making your selection.

☒ Life sciences ☐ Behavioural & social sciences ☐ Ecological, evolutionary & environmental sciences

For a reference copy of the document with all sections, see nature.com/documents/nr-reporting-summary-flat.pdf

Life sciences study design

All studies must disclose on these points even when the disclosure is negative.

Sample size	These experiments were not designed to compare an intervention to a control; thus, statistical considerations such as sample size, randomization, and blinding were not performed. The hypothesis for the guinea pig transmission experiments (Fig. 3) was that influenza virus transmission by aerosolized fomites is biologically possible (i.e., that the probability of transmission by aerosolized fomites is non-zero). The hypothesis requires no statistical inferences to be made, and no conclusions were drawn about the probability of transmission under these conditions, beyond establishing a non-zero transmission probability; thus, a priori power calculations using frequentist statistical methods were not performed. Bayesian methods were used a posteriori to estimate a 95% credible interval for the transmission probability. The other experiments (Figs. 1, 2, and 4) were non-hypothesis-driven, and no formal hypothesis-testing was performed. Data are descriptive, and all data are included in the figures.
Data exclusions	No data were excluded.
Replication	Fig. 1: Three biological replicates (three individual guinea pigs) were measured for each experimental condition variable. Each APS experiment was performed once per condition (one technical replicate of each biological replicate). For the awake, mobile guinea pigs, the variable experimental condition was bedding type (3 different bedding types, one 1-hour measurement per bedding type per guinea pig). For stationary guinea pigs, the variable in the experimental conditions were pre-infection vs. post-infection with Pan99 virus and anesthetized vs. euthanized guinea pigs. Measurements on anesthetized guinea pigs were performed on 4 different days (pre-inoculation and days 1, 2, and 3 post-inoculation, one 30-minute measurement per day per guinea pig), and once with the euthanized guinea pigs (one 30-minute measurement per guinea pig).<br>Fig. 2: Two biological replicates (two individual guinea pigs) were performed. One swab per area (fur, ears, paws, and cages) was taken, and one plaque assay per swab eluate was performed (one technical replicate per swab from each biological replicate).<br>Fig. 3: Three replicate sets of 4 transmission pairs (1 virus-donor and 1 virus-recipient guinea pig per pair, 12 pairs total) were performed, with transmission rates of 1/4, 2/4, and 0/4 in each replicate. Thus, 2 of the 3 replicates successfully confirmed the hypothesis that the probability of transmission by aerosolized fomites is non-zero.<br>Fig. 4: Fig. 4b was performed once; all data are shown (including in Supplementary Movie 2). For Fig. 4c, one biological replicate of the positive control was performed, with two technical replicates (plaque assays) from the biological replicate. Two biological replicates of the negative control were performed, with one technical replicate (plaque assay) from each biological replicate. Two biological replicates were performed with lab wipe and paper towel. Two technical replicates (plaque assays) were performed from each biological replicate. Two biological replicates were planned for toilet paper, but only one was performed because the paper disintegrated during manipulation. Two technical replicates (plaque assays) were performed from the one biological replicate.<br>Supplementary Figs. 1, 2, and 3a-3c: Representative data from the experiments shown in Fig. 1, as described above.<br>Supplementary Fig. 3d: Representative data from one biological replicate of the experiment shown in Fig. 4b, as described above.<br>Supplementary Fig. 6: Swabs were taken during two of the three transmission experiment replicates represented in Fig. 3. One swab per area (fur, ears, paws, and cages) was taken per guinea pig per time point, and one plaque assay was performed from each swab eluate.<br>Supplementary Fig.7: Representative data from the experiments shown in Fig. 4c, as described above.
Randomization	Figs. 1 and 2: Guinea pigs were selected from cohousing cages randomly, but formal randomization of guinea pigs was not performed.<br>Fig. 3: Donor guinea pigs and recipient guinea pigs were cohoused separately (donors with donors and recipients with recipients) prior to each experiment. Animals were taken randomly from cohousing cages to create transmission pairs for the experiment, but formal randomization/allocation of guinea pigs into transmission pairs was not performed.
Blinding	Figs. 1 and 2: These experiments were non-hypothesis-driven, and resultant data are descriptive. The investigators were not blinded to the interventions that were being performed.<br>Fig. 3: Animals were taken randomly from cohousing cages to create transmission pairs for the experiment, but investigators were not blinded as to the intervention (virus infection), which all animals received. Results are compared to historical data; no control group was included to reduce animal usage.

Reporting for specific materials, systems and methods

We require information from authors about some types of materials, experimental systems and methods used in many studies. Here, indicate whether each material, system or method listed is relevant to your study. If you are not sure if a list item applies to your research, read the appropriate section before selecting a response.

Materials & experimental systems

n/a	Involved in the study
☒	☐ Antibodies
☐	☒ Eukaryotic cell lines
☒	☐ Palaeontology
☐	☒ Animals and other organisms
☒	☐ Human research participants
☒	☐ Clinical data

Methods

n/a	Involved in the study
☒	☐ ChIP-seq
☒	☐ Flow cytometry
☒	☐ MRI-based neuroimaging

Eukaryotic cell lines

Policy information about cell lines

Cell line source(s)	MDCK-SIAT1 cells were purchased at passage (P-)6 from the European Collection of Authenticated Cell Cultures (ECACC) through Millipore Sigma USA (SKU #0507-1502, lot #15B002). Cells were thawed and expanded by serial subculture in G418 selective medium, as per ECACC instructions. One-millilitre aliquots of 10^7 cells suspended in heat-inactivated Fetal Bovine Serum (FBS) supplemented with 10% dimethylsulfoxide (DMSO) were frozen down at P-9 in liquid nitrogen. P-9 aliquots were thawed and passaged by subculture to perform virus titrations. Cells were replaced by a new P-9 aliquot prior to P-30.
Authentication	MDCK-SIAT1 cells ECACC lot #15B002 were authenticated by DNA bar-coding sequencing of the mitochondrial cytochrome c oxidase gene (SOP ECC5), test #53175 on 17/03/2015.
Mycoplasma contamination	MDCK-SIAT1 cells ECACC lot #15B002 were confirmed mycoplasma-free by Mycoplasma DNA PCR (SOP ECC73) and by Hoechst 33258 fluorescent detection assay in a Vero indicator cell line (SOP ECC137), test #53175 on 06/03/2015.
Commonly misidentified lines (See ICLAC register)	MDCK cells are not listed in ICLAC Register Version 9.

Animals and other organisms

Policy information about studies involving animals; ARRIVE guidelines recommended for reporting animal research

Laboratory animals	Guinea pig (Cavia porcellus), Hartley strain, females. At the time of the experiments, guinea pigs were 5-6 weeks old (400-450 g) with the exception of the previously infected, Pan99-immune guinea pigs in the contamination transmission experiments, which were 12-16 weeks old (700-800 g) at the time.
Wild animals	No wild animals were used in the study.
Field-collected samples	No field-collected samples were used in the study.
Ethics oversight	All procedures were performed in strict accordance with the recommendations in the Guide for the Care and Use of Laboratory Animals (8th edition, 2011) and the AVMA Guidelines for the Euthanasia of Animals (2013), which were in force at the time of these experiments. The research protocol and all subsequent amendments were approved by the Icahn School of Medicine at Mount Sinai Institutional Animal Care and Use Committee (IACUC protocol #2014-0178).

Note that full information on the approval of the study protocol must also be provided in the manuscript.
